# Transcriptomic analyses reveal that the cellular Gem protein promotes HTLV-1 infected cell migration and viral transmission

**DOI:** 10.1186/1742-4690-11-S1-O64

**Published:** 2014-01-07

**Authors:** Sébastien A Chevalier, Cynthia A Pise-Masison, Antoine Gessain, Renaud Mahieux

**Affiliations:** 1 Oncogenèse Rétrovirale, label “Ligue Nationale Contre le Cancer”, CIRI, LabEx ECOFECT, INSERM U1111 - CNRS UMR 5308, Ecole Normale Supérieure - Université Lyon 1, Lyon, Cedex 07, France; 2 Animal Models and Retroviral Vaccine Section, Vaccine Branch, CCR, National Cancer Institute, National Institutes of Health, Bethesda, MD, USA; 3 Epidémiologie et Physiopathologie des Virus Oncogènes, CNRS UMR 3569, Pasteur Institute, Paris, Cedex 15, France

## 

In a previous study, we used gene expression microarrays and functional assays to identify cellular genes whose expression profiles were similarly affected by Tax proteins from all three HTLV subtypes (HTLV-1, HTLV-2 and HTLV-3). We found forty-eight genes up-regulated by all three Tax proteins (Chevalier *et al*, *Plos One*, 2012). Among those, *Gem*, which encodes a member of the Ras GTP-binding proteins superfamily, was strongly up-regulated. Herein, we first show that *Gem* expression is strongly up-regulated at the protein level not only in Tax-expressing cells, but also in all tested HTLV-infected cell lines and in primary uncultured T lymphocytes isolated from TSP/HAM patients. We then demonstrate that Tax activates transcription from the Gem promoter through the recruitment of CREB and CBP/p300 onto a cAMP Responsive Element (CRE). Gem protein has been shown to regulate reorganization of the cell cytoskeleton. Since efficient transmission of HTLV-1 from infected to uninfected T cells is mediated by cell-cell contacts, whose formation relies on cytoskeletal reorganization, we investigated the impact of Gem expression on cell migration and formation of cell-cell contacts. Our results show that Gem-overexpressing T lymphocytes display an increased spontaneous migration, while Gem-knocked down HTLV-infected cell lines show a strong reduction in their ability to migrate. We also observe that Gem enhances conjugate formation between infected and non-infected T lymphocytes. Altogether, our results indicate that Gem could be essential for the cell-to-cell spread of HTLV.

